# Supplementary material for: Direct Photochemical Synthesis of Substituted Benzo[b]fluorenes
Source: Org Lett. 2024 Nov 25;26(48):10364–8. doi: 10.1021/acs.orglett.4c03978 (PMC11629382; doi:10.1021/acs.orglett.4c03978)
Supplement: Supplementary file 2 — ol4c03978_si_002.zip [file ol4c03978_si_002.zip › FID for Publication/5e/20240610_RC-2-C-5-98B-P2_01/plots/RC-2-C-5-98B-P2_gHMBCAD_20240610_01_plot01.pdf]

RC-2-C-5-98B-P2

Location **15** | Pulse sequence **gHMBCAD** | Temperature **25** | Suboperator **RuairiCrawford**
Date collected **2024-06-10** | Solvent **dmso** | Spectrometer **localhost.localdomain-vnmrs500** | Operator **MBaumann**

SAMPLE

date	Jun 10 2024
solvent	dmso
sample	20240610_RC-2-C-5-98B-P2_01

ACQUISITION

sw	6410.3
at	0.150
np	1924
fb	4000
ss	32
d1	1.000
nt	8

2D ACQUISITION

sw1	30154.5
ni	256
phase	arrayed

PRESATURATION

satmode	n
wet	n

TRANSMITTER

tn	H1
sfrq	499.724
tof	161.2
tpwr	58
pw	6.300

DECOUPLER

dn	C13
dof	1285.1
dm	nnn
decwave	W40_P7374
dmf	32258
dpwr	43
pwxlvl	62
pwx	14.000

HMBC

j1xh	146.0
jnxh	8.0

ADIABATCIC

pwx180ad	P7374_ad300
pwxlvl180	57
pwx180	465.4

FLAGS

hs	nn
sspul	y
PFGflg	y
hsglvl	5736

SPECIAL

temp	25.0
gain	42
spin	0

GRADIENTS

gzlvl1	478
gt1	0.001000
gzlvl3	1434
gt3	0.001000
gstab	0.000500

F2 PROCESSING

sb	-0.075
sbs	not used
fn	2048

F1 PROCESSING

gf1	0.008
gfs1	not used
proc1	lp
fn1	2048

DISPLAY

sp	-545.3
wp	6410.3
sp1	-1883.5
wp1	30154.5
rfl	545.3
rfp	0
rfl1	1883.5
rfp1	0

PLOT

wc	142.6
sc	0
wc2	127.1
sc2	12.7
vs	231
th	3
ai	cdc av

ACQUISITION ARRAYS

array	phase
arraydim	512
i	phase
1	1
2	2

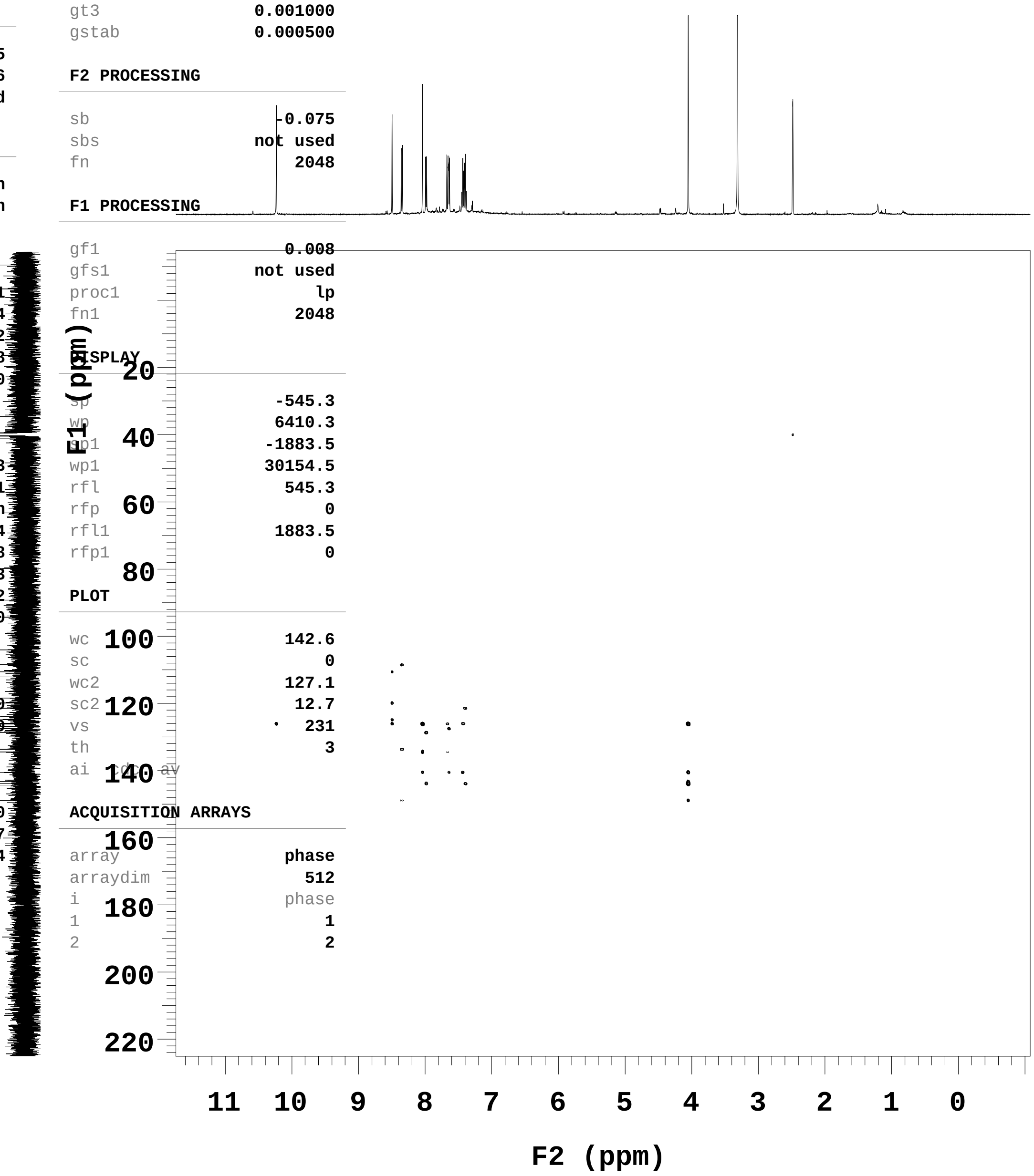

Plotname: RC-2-C-5-98B-P2_gHMBCAD_20240610_01_plot01

Data file /home/data/MBaumann/cscb500/RuairiCrawford/2024/20240610_RC-2-C-5-98B-P2_01/RC-2-C-5-98B-P2_gHMBCAD_20240610_01.fid Plot date 2024-06-10
